# On the Role of the Electrical Field in Spark Plasma Sintering of UO_2+x_

**DOI:** 10.1038/srep46625

**Published:** 2017-04-19

**Authors:** Vaclav Tyrpekl, Mohamed Naji, Michael Holzhäuser, Daniel Freis, Damien Prieur, Philippe Martin, Bert Cremer, Mairead Murray-Farthing, Marco Cologna

**Affiliations:** 1 European Commission, Joint Research Centre (JRC), Postfach 2340, 76125 Karlsruhe, Germany; 2 CEA, DEN, DTEC, Centre d’études nucléaires de Marcoule, Bagnols-sur-Cèze F-30207, France

## Abstract

The electric field has a large effect on the stoichiometry and grain growth of UO_2+x_ during Spark Plasma Sintering. UO_2+x_ is gradually reduced to UO_2.00_ as a function of sintering temperature and time. A gradient in the oxidation state within the pellets is observed in intermediate conditions. The shape of the gradient depends unequivocally on the direction of the electrical field. The positive surface of the pellet shows a higher oxidation state compared to the negative one. An area with larger grain size is found close to the positive electrode, but not in contact with it. We interpret these findings with the redistribution of defects under an electric field, which affect the stoichiometry of UO_2+x_ and thus the cation diffusivity. The results bear implications for understanding the electric field assisted sintering of UO_2_ and non-stoichiometric oxides in general.

Spark Plasma Sintering (SPS) is one of the state of the art techniques for the facile consolidation of virtually all ceramic and metallic powders^1^,^2^,^3^,^4^,^5^. The combination of pressure, rapid heating and possible activating effect of electric field, allows the creation of materials with new microstructures and new properties, with significant time and energy saving compared to the well-established traditional sintering mass production path. Although SPS is now a common tool in materials research, its application to nuclear fuels has not been explored extensively, possibly due to complexity associated with the safe handling of actinides. Recent research in Electric Field Assisted Sintering (EFAS) of actinide oxides and nitrides has shown the great potential of the technique, but has also evidenced the need for a better understanding of the behaviour of actinides during EFAS^6^,^7^,^8^,^9^,^10^,^11^,^12^,^13^,^14^. Uranium oxide, due to its multiple oxidation states and electrical characteristics, is an ideal model material to investigate some of the peculiar effects of the electric field in the processing of ceramic materials.

For nuclear energy applications, the possible deviation from stoichiometry of UO_2_ is of paramount importance, since it governs its safety relevant properties, *inter alia* the thermal conductivity, the mechanical properties, the diffusion coefficients etc. The stoichiometry has to be strictly controlled during synthesis. The conditions for the control of the O/U ratio are well known for the traditional sintering process: one typical route to obtain UO_2.00_ pellets consists in sintering hyperstoichiometric (UO_2+x_) powder in an Ar/H_2_ gas mixture for several hours at temperatures around 1700 °C.

In case of Spark Plasma Sintering of UO_2+x_, the suitable sintering and reduction conditions are more complex to identify; the oxygen potential of the gas in the die cavity is not well defined and can differ substantially from the external set gas atmosphere: the reaction of graphite from the tools with the oxygen coming from the sample or from impurities creates CO gas and thus a reducing atmosphere, whose oxygen potential is hard to evaluate^3^. The external field and high current passing in the die/green body assembly may also play a role, which is still far from being fully understood.

Ge *et al*.^7^ studied the evolution of the oxygen to metal (O/M) ratio of UO_2+x_ in SPS. It was found that the stoichiometry decreases monotonically with the maximum hold time and temperature, and an O/M ratio of 2.00 can be achieved at 1450 °C with a 0.5 min hold time under inert gas conditions. These findings were explained by a solid state reaction of the excess oxygen in UO_2+x_ with the graphite of the tools. The O/M ratio was determined as the average value for the whole pellet, and no information on local variations was obtained. The possible effect of the electric field was also not discussed.

Reduction effects in SPS are known also for other materials; Anselmi-Tamburini *et al*.^15^,^16^ found that the colour of Yttria Stabilised Zirconia (YSZ) turned from white to grey to black when the sintering temperature was increased above 1200 °C. Detailed investigations excluded carbon contamination, and the coloration was attributed to oxygen deficiencies. In certain cases also a colour gradient in the axial direction was observed, which was thought to be the consequence of gradient in stoichiometry resulting from the axial gradient in temperature due to the non-perfect centring of the sample in the tools. Valenzuela *et al*.^17^ reported the partial reduction of Fe^3+^ to Fe^2+^ during SPS of NiZn ferrites of composition Zn_0.7_Ni_0.3_Fe_2_O_4_. In both cases^15^,^17^, the reduction was attributed to a chemical reaction with graphite.

The role of the electrochemical reactions at the electrodes was recognised by Beekman *et al*.^18^, who demonstrated a novel solid state reaction route for the formation of a Na_24_Si_136_ clathrate phase starting from sodium silicide. The process is driven by the pulsed DC electric field and is thus asymmetrical: the clathrate is formed starting from the anode by oxidation of silicon ions, while Na is formed and evaporated at the cathode.

In this work we investigate the effects of the electric field on the reduction of UO_2+x_ to UO_2_ during SPS processing, by combining X-ray diffraction (XRD), X-ray absorption spectroscopy (XAS), micro-Raman spectroscopy, microstructural investigation and electro-thermal modelling. We found that the electrical field in SPS plays a significant role in the reduction of UO_2+x_. Pellets sintered at low temperatures show a gradient in the oxidation state, and the oxidation state depends unequivocally on the direction of the field. The electric field influences also the grain growth rate of UO_2+x_, most likely by controlling the defect redistribution of the oxygen interstitials.

## Results

### Average stoichiometry

**Figure 1:**
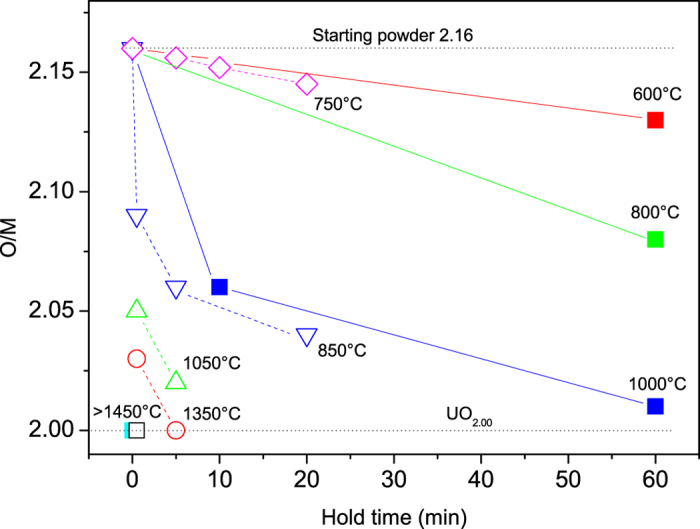
Average O/M ratio of UO_2_ disks consolidated by Spark Plasma Sintering as a function of holding time at the maximal temperature, for different maximal temperatures. Open symbols are from Ge *et al*.^7^, solid points from this work. The lines between the data points are only a guide for the eyes.

The average stoichiometry of the sintered disks as a function of the maximum processing temperature and dwell time is given in Fig. 1. The open symbols are from Ge *et al*.^7^ and were assessed by the weight change of the pellets after full reduction to UO_2.00_ in a conventional furnace; the solid points are from this work, where the O/M ratio is evaluated from the shifts of the XRD pattern of the sintered disk after being pulverised in a mortar.

Upon sintering in SPS a reduction is always observed. The decrease in the O/M ratio is time and temperature dependent; for a hold temperature of 1000 °C the time to reach O/M of 2.00 is more than 1 h, whereas at temperatures higher than 1450 °C, only 0.5 min dwell time gives complete reduction to UO_2.00_. The data presented herein confirm the general trend reported in Ge *et al*.^7^; the discrepancies in the values between the two set of experiments can be attributed to differences in the boundary conditions, *i.e.* different die geometries, starting powder, heating rates, pressures, etc., and to the different set-ups used: in Ge *et al*.^7^ the temperature was measured on the outer surface of the die (Sumitomo SPS design), while in our experiments, the temperature was measured by a thermocouple placed in the cavity of the lower graphite piston, very close to the sample (FCT SPS design).

### Charge distribution (XANES)

**Figure 2:**
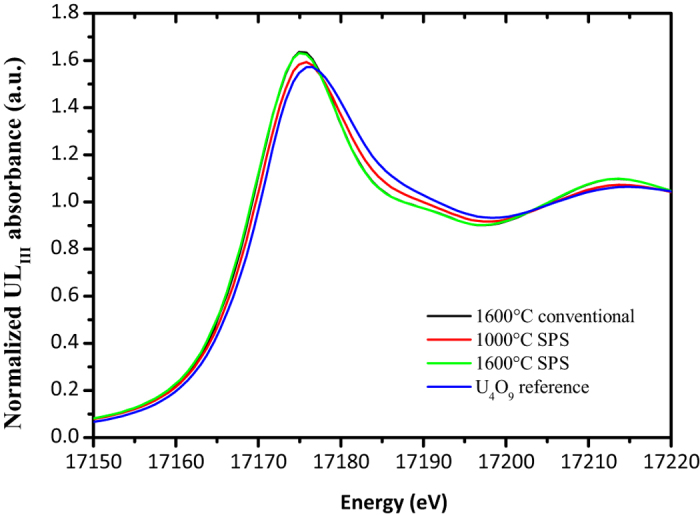
U LIII XANES spectra of samples sintered in SPS at 1000 °C and 1600 °C for 5 min, conventionally at 1600 °C for 4 h and of a reference U_4_O_9_.

The charge distribution of the field assisted sintered pellets was evaluated by XAS and compared to conventionally sintered UO_2_ as a reference. The U LIII XANES spectra of chips taken from the inner volume of samples processed in SPS at 1000 °C and 1600 °C are presented in Fig. 2, along with the reference spectrum of U_4_O_9_; the results of the analysis are summarised in Table 1. For a processing temperature of 1000 °C and 5 minutes hold time, the reduction to UO_2.00_ is not complete. The U(V) content of the analysed chip from the central zone of the pellet is 24% and the calculated O/M is thus 2.12.

The spectra of UO_2_ sintered conventionally and in SPS at 1600 °C are identical; in both cases only U(IV) is detected and the O/M is 2.00. This confirms that SPS can produce structurally identical UO_2_ just as conventional sintering. The XANES analysis does not reveal any noticeable influence of the electric field in the charge distribution of UO_2.00_ and in the formation of anomalous structural defects.

### Stoichiometry near the positive and negative contact surfaces

**Figure 3:**
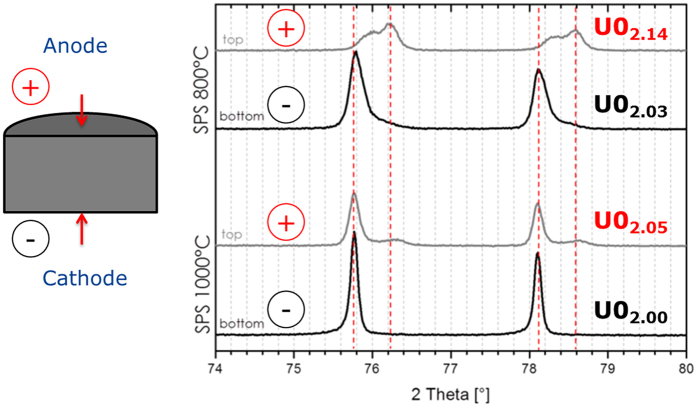
Detail of the X-ray diffraction pattern of positive and negative surfaces of UO_2_ pellets sintered in SPS at 800 °C and 1000 °C for 5 minutes, starting from hyperstoichiometric powder.

The O/M ratios shown in Fig. 1 represent an average of the oxidation state of the disks, and thus do not reveal any possible local variation, or potential effects of the external electric field. The upper and lower surfaces of two pellets sintered in SPS at temperatures of 800 °C and 1000 °C for 5 min, were measured by XRD, in order to investigate the oxidation state of the surfaces and its relation to the direction of the electric field. The penetration depth of the X-rays in the samples is only in the micrometer range, and thus the oxidation state of the region very close to the electrodes (upper and lower pistons of the die) can be assessed. In the SPS configuration used, the upper piston is the positive electrode. The most significant results are shown in Fig. 3 and summarised in Table 2. The surfaces of the sample sintered at 1000 °C have generally a lower content in oxygen compared to those of the sample sintered at 800 °C, consistently to the data presented in Fig. 1. But, most interestingly, the positive surface shows always a higher oxidation state compared to the negative one.

### Axial and radial stoichiometry gradients

**Figure 4:**
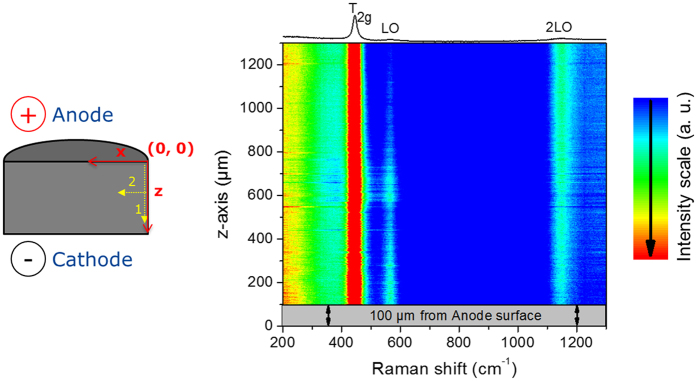
Raman mapping along the axial direction (along the dotted arrow 1 from z = 100 μm to 1300 μm at x = 80 μm) on the cross section of a pellet sintered at 1000 °C for 5 min.

The presence of an axial and radial oxidation gradient within the bulk of the pellet was investigated by micro-Raman spectroscopy. This technique is highly sensitive to the stoichiometry variations in UO_2+x_^19^. The intensity ratio of the peak at 575 cm^−1^ (LO peak) to the one at 1550 cm^−1^ (2LO peak) is in fact proportional to the O/M ratio. More information on this subject is given in Appendix 2. Scans were performed along the axial (z-axis) and radial (x-axis) directions, along the dotted arrows 1 and 2 (Fig. 4), as explained in the experimental section.

Figure 4 displays a Raman map performed on the axial direction of an axial cross section up to a z value of 1300 μm. The map consists of a colour map, with Raman shift scale as abscissa and depth position as ordinate. The colour is linked to the Raman intensity; red is assigned to the highest intensity.

**Figure 5:**
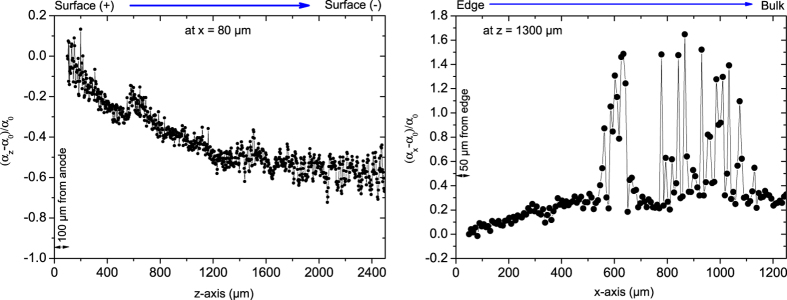
Evolution of the relative intensity ratio *α* = I(LO/2LO) along the axial (**a**) and radial (**b**) direction. The scans are taken along the arrows 1 and 2 as indicated in Fig. 4.

Figure 5(a) and (b) show the relative evolution of the ratio *α* = I(LO/2LO) along the axial and radial directions, respectively. In Fig. 5(a), the intensity ratio decreased almost linearly up to a z-depth of about 500 μm from the anode then it stabilises at minimum value near the central region. Beyond the central zone, the intensity ratio is almost constant, with a slight drop when approaching the cathode region. It is interesting to note that the relative change in *α* ratio is about 60% between the surface and the centre. The measurement indicates a decrease of the oxidation state from the anode surface to the centre (note that the measurement is taken along a line close to the outer radius of the pellet, at x = 80 μm). Figure 5(b) shows an increase of *α* when moving from the outer radius to the centre. It is interesting to note the appearance of highly-oxidized points (with very high *α* ratio) when approaching the central zone of the pellet. These points correspond to Raman spectra very close to that of U_4_O_9_ structure^20^, and thus suggest the presence of very small regions with highly oxidised phases. It is well known that individual band analysis (as made in the section above) can lead to ambiguous conclusions regarding the evolution of the Raman intensities. Therefore, component analysis was performed on the axial set of data. This is shown in Appendix 2 and confirms the trend observed in Fig. 5.

The Raman spectra give thus the following picture: (i) in the axial direction the O/M ratio is decreasing while approaching the centerline of the pellet from the anodic surface (Fig. 5a), when moving on a line very close to the pellet outer radius, and (ii) in the radial direction the O/M ratio is increasing while approaching the central zone (Fig. 5b). The data suggest thus the presence of two regions of higher oxidation state: a region close to the anode and the central region of the pellet.

### Microstructural gradients

**Figure 6:**
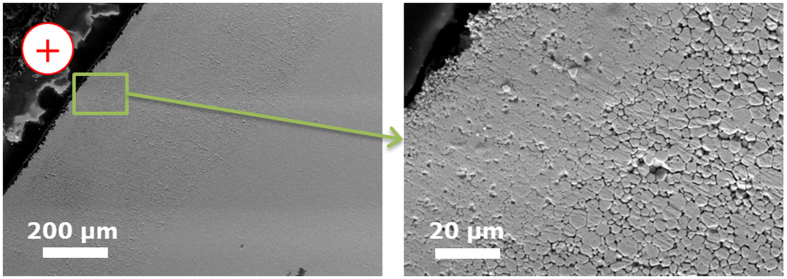
Microstructure of the area close to the anode of a pellet sintered at 1200 °C for 5 min in SPS.

**Figure 7:**
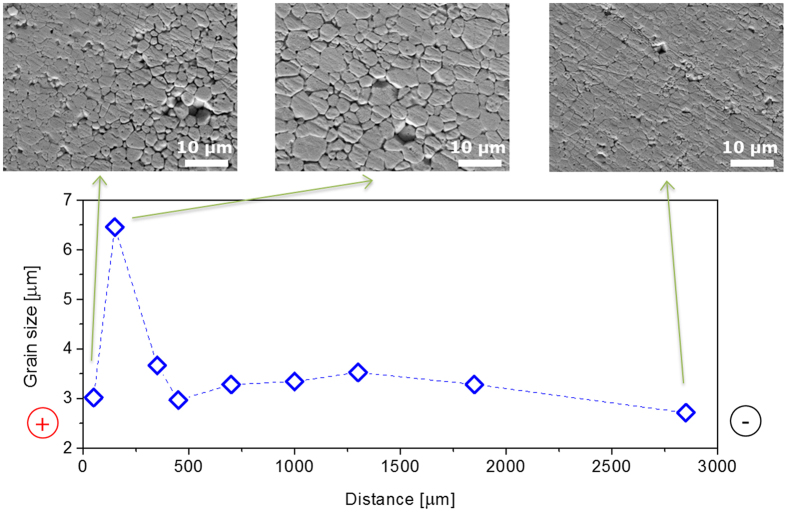
Grain size as a function the distance from the anode of a pellet sintered for 5 min at 1200 °C in SPS, measured in the centre of the pellet. The line connecting the data points is only a guide for the eyes.

The microstructure of a pellet sintered at 1200 °C for 5 min is shown in Figs 6 and 7 and the grain size as a function of the distance from the cathode is shown in Fig. 7. The microstructure reveals a very peculiar feature: a region with larger grain size. Its location is close to the positive piston, but not in contact to it. In this region the grain boundaries are also more visible, possibly due to more effective chemical etching. The grain size shows a maximum of 6.5 μm at a distance of around 150 μm from the positive electrode. In the other regions, the grain size is less variable, but a second local maximum of ~3.5 μm can be observed in the central area of the pellet, at a distance of 1300 μm from the positive electrode (Fig. 7).

### Temperature homogeneity

**Figure 8:**
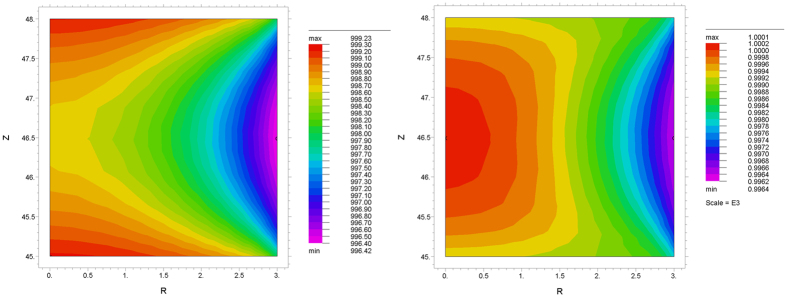
Calculated temperature distribution at a nominal sintering temperature of 1000 °C for 3 mm thick dense pellets of UO_2.00_ (left) and UO_2.16_ (right).

The temperature and current distribution during sintering were assessed by FEM simulations. The temperature distribution for a nominal sintering temperature of 1000 °C and 3 mm disk height for the two limiting stoichiometries of UO_2.00_ and UO_2.16_ is shown in Fig. 8. A clear difference in the temperature distribution within the pellet is observed between UO_2.16_ and UO_2.00_, caused by the higher electrical conductivity of the oxidised phase. However, the temperature gradients are always very contained and never higher than 4 °C. The temperature differences at lower nominal temperatures and for 1 mm disk height are even lower. For the nominal temperature of 1600 °C the temperature difference within the disk is still below 9 °C and 4 °C for UO_2.16_ and UO_2.00_, respectively (3 mm pellet height).

An additional potential source of temperature inhomogeneity is the heating or cooling at the electrodes/sample interfaces due to the Peltier effect, which becomes noticeable when a significant amount of current passes through the interface of materials with large difference in the Peltier coefficients, and in some cases can account for up to 10% of the total dissipated heat during SPS^3^,^21^. The results of the FEM simulations (Appendix 1) show that the current is flowing mostly through the surrounding graphite die. The calculated Peltier power for disks and pellets at 800 °C and 1000 °C is never larger than 1 W, and thus the effect on the temperature distribution is considered to be negligible.

## Discussion

Hyperstoichiometric uranium dioxide powder sintered in SPS at 1600 °C is reduced to stoichiometric UO_2.00_. The obtained pellets are structurally identical to the reference pellets conventionally sintered in Ar/H_2_, as confirmed by XANES. In conventional sintering, a reducing processing gas (typically Ar/H_2_ mixtures) is used to achieve reduction to UO_2.00_. In the case of SPS instead, the conditions can be sufficiently reducing to reach stoichiometric UO_2_, even in the absence of H_2_.

However, our results point out to the presence of a gradient in the oxidation state for pellets sintered in SPS at lower temperatures, where the reduction is not completed to UO_2.00_. The oxygen distribution and the average stoichiometry vary and depend on sintering time and temperature; in general, the O/M ratio is lower at the surfaces in contact with graphite and higher in the centre of the pellet. But the oxygen distribution is not symmetrical with respect to the central radial section: the surface in contact with the positive piston shows a higher oxygen content than the negative side (Fig. 3)

The lower oxidation state of the surfaces compared to the centre can be due to the graphite surrounding the pellet. The reduction of UO_2+x_ to UO_2_ has been explained by the reduction of the oxide with the graphite, according to Eq. 1^7^.


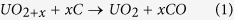


The CO gas produced in this reaction can further act as a reducing agent for UO_2+*x*_. If the reaction happens at the die surfaces, a gradient in the oxidation state of the pellet during the intermediate stages of the process is to be expected. If this would be the sole cause of oxygen redistribution, the stoichiometry would show a maximum in the centre of the pellet and a minimum at the surfaces in contact with the die, and would be symmetrical along the axial and radial directions.

Thus the effect of the graphite alone cannot account for the non-symmetrical distribution of the O/M ratio. Sources of asymmetry in the axial direction could in principle be twofold: (i) the electric field, and (ii) thermal gradients due to Peltier effect. FEM simulations showed a rather homogeneous temperature distribution and excluded significant contribution of Peltier heating and cooling. Thus, we conclude that the oxygen redistribution in the vertical direction is to be attributed solely to the effect of the electric field, which drives the negatively charged oxygen ions towards the positive electrode. These two factors (reduction starting from the pellet surfaces and asymmetrical oxygen distribution due to electric field) superimpose and create a complex spatial distribution of the O/M ratio.

As a further confirmation, the microstructure of the pellets shows a memory of the local stoichiometry during sintering. The oxidation state of U in UO_2+x_ has a direct impact on the microstructure of the pellets. In general in oxides the electrical ionic conductivity is controlled by the mobility of the fastest moving species, whereas the rate of the neutral mass diffusional processes (as creep, sintering, grain growth) are governed by the transport of charge neutral molecules whose diffusion rate is controlled by the slowest moving charged species^22^. In the UO_2+x_ lattice, the self-diffusion of oxygen anions is faster than self-diffusion of the uranium cations^23^,^24^. The redistribution of oxygen subjected to the external electric field is governed by the oxygen diffusivity. But the rate of sintering and grain growth depends on the self-diffusivity of U in UO_2+x_, which in turn is a function of the O/M ratio^24^, being higher at higher O/M ratios. The area with larger grains in proximity to the anode (Fig. 7), is most likely the area that was at the highest oxidation state (and thus with highest U self-diffusion coefficient), at the time when a microstructure with close porosity was reached and grain growth started to be significant.

The presence of areas with larger grains at the anode was observed also by Zhang *et al*.^25^ in the flash sintering of ZnO, and by Kim *et al*.^26^ for YSZ, at the cathode. In both cases the larger grain zone was at the interface with the electrode and not inside the bulk of the material, as in the present case. The proposed explanations were electric-potential-induced accumulation of electrons and an associated oxidation reaction to form excess cation vacancies at grain boundaries that promote interfacial diffusion^25^, as well as electric-potential-induced abnormal grain growth^26^, and grain boundary complexion transitions^27^.

Rheiheimer *et al*.^28^ recently conducted a model experiment to study the effect of weak electric fields on grain growth in strontium titanate. The observed enhanced grain boundary mobility at the negative electrode was attributed to the migration of the charged defects induced by the electric field (oxygen vacancies towards the negative electrode and strontium vacancies towards the positive electrode). The grain growth rate in strontium titanate is in fact strongly dependent on the defect chemistry, as in the case of UO_2_. In agreement with^28^, we suggest that also in the case of UO_2+x_ the area with coarsened grains is to be attributed to the higher rate of grain growth due to the higher stoichiometry (and thus grain boundary mobility) during processing.

The exact location of the area at higher stoichiometry results from the balance between the electric field, which drives the oxygen ions towards the positive electrode and the reducing effect of the graphite at the pellet surfaces. The phenomena observed in UO_2+x_ could be more general, and common to other non-stoichiometric oxides, and may contribute to elucidate some of the electric field induced peculiarities that are often observed in the materials processing techniques assisted by DC fields, such as SPS and Flash Sintering^5^,^29^.

In order to increase the microstructural homogeneity of the pellets, different technical solutions can be proposed, for example the use of pre-reduced UO_2_ powder (eventually at the expense of sinter activity), the use of AC fields instead of DC, the electrical insulation of the sample from the graphite pistons through the use of specific separators, or the use of lower heating rates, allowing the powder to be reduced to UO_2.00_ before full sintering and subsequent rapid grain growth can take place.

## Conclusions

UO_2+x_ powders sintered in SPS are gradually reduced to a lower stoichiometry as a function of temperature and time. Powders sintered in SPS at 1600 °C give dense pellets that are structurally identical to the conventionally sintered ones in Ar/H_2_. Powders sintered at conditions for which the reduction is not complete, show a gradient in the oxidation state. The shape of the gradient is given by two superimposing effects: (i) the reducing effect of the carbon environment, and (ii) the direction of the electrical field, which is driving oxygen defects towards the anode.

The microstructure shows a memory of the history of the O/M gradient: zones that have experienced a higher oxidation state at the time and temperature after which densification occurred show a larger grain size. The proposed role of the electrical field in this study is to redistribute the oxygen defects and thus to change the local cation diffusivity, which governs sintering rate and grain growth rate. These observations are likely to be not limited to UO_2+x_ but common to other non-stoichiometric oxides, and may contribute to shed some light on the poorly understood and highly disputed role of the electric field in EFAS techniques such as SPS and Flash Sintering.

## Methods

All the preparations and analysis were performed in hermetically sealed gloveboxes under a protecting Ar or N_2_ atmosphere, with the exception of the micro-Raman analysis and the manual grinding of the powders for XAS experiments, which were conducted in ambient air. The starting material was a commercial UO_2_ powder obtained from the Ammonium Di-Uranate (ADU) route, with primary particle size of ca. 200 nm, and an O/U ratio of 2.16, as evaluated by XRD.

Spark plasma sintering was conducted in an SPS (FCT Systeme GmbH, Rauenstein, Germany) installed in a glovebox for handling radioactive substances^6^. Both glovebox and SPS chamber were kept under Ar atmosphere. The UO_2_ powder was loaded directly in 6 mm diameter graphite dies, without using any graphite separation paper. About 300 mg of powder was used for making short pellets (later on referred as “disks”), and 900 mg for taller pellets. The heating and cooling rates were 200 °C/min and uniaxial pressure of 70 MPa was applied constantly from room temperature. The maximum temperature and dwell time were varied.

XRD patterns were measured on a Brucker D8 diffractometer with a Bragg–Brentano (θ/2θ) geometry, with a Kα1 monochromator and a Lynxeye detector, in the 2θ range of 10°–120°, with steps of 0.013°. The O/U in UO_2+x_ was determined from the lattice parameter (a, in Å) obtained from the Rietveld refined patterns, according Eq. 2^30^:


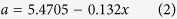


The X-ray absorption near edge structure (XANES) measurements were performed at the European Synchrotron Radiation Facility (ESRF, Grenoble, France) on the Rossendorf Beamline (BM20) with a current of 170–200 mA in the storage ring (at 6.0 GeV)^31^. The XANES spectra were measured at the U L_III_ (17166 eV) edge in transmission mode. A double Si (111) crystal monochromator was used for energy selection and the energy calibration was performed using Y metallic foils (17038 eV). Data analyses and refinements were performed using the *Athena* program^32^,^33^. XANES spectra were normalized using a linear function and a 2^nd^ order polynomial for pre- and post-edge approximation, respectively. The first zero crossings of the first and second energy derivatives were used to determine the white line (WL) and inflection point (E_0_) energy positions, respectively. Average oxidation states of the cations were determined by Linear Combination Fitting (LCF) of the experimental normalized absorption spectra by using well-known reference spectra. The reference compounds used were U^+IV^O_2.00(1)_^34^, (U^+IV^_½_U^+V^_½_)_4_O_9_^34^ and (U^+V^_⅔_U^+VI^_⅓_)_3_O_8_^35^. The XANES spectra of the reference compounds have been recorded previously at the same beamline. The LCF region is [−20;30 eV] relatively to the WL position. The uncertainty for the determined molar fractions is 2 mol.% and for O/M ratio is 0.01. The XANES spectra were collected on the following samples: (i) a disk sintered conventionally for 4 h at 1600 °C in Ar/H_2_, (ii) a chip from a disk sintered for 5 min at 1600 °C in SPS in Ar, and (iii) a chip from the central region of a pellet sintered in SPS for 5 min at 1000 °C.

Micro-Raman line scan measurements were performed at room temperature on a 6 mm diameter and 3 mm height UO_2_ pellet sintered in SPS at 1000 °C for 5 minutes. The pellet was cut into two half-disks to reveal the axial cross section and polished. The laser power at the sample surface was kept below 3 mW to avoid any undesirable heat and oxidation of the sample surface. The optical image and the Raman analysed image were calibrated using a silicon integrated circuit. Micro-Raman spectra were collected using a Horiba Jobin-Yvon T 64 000 spectrometer equipped with 647 nm of Kr+ excitation laser and a 1800 grooves/mm grating allowing acquisition from 200 to 1300 cm^−1^. An objective of x100 magnification with a numerical aperture of 0.85 was used, which gives a spatial resolution of better than 1 μm. Raman mapping was performed along the axial (z) and radial (x) direction of the pellet. The origin of the coordinate system (x = 0, z = 0) was defined as the top right corner of the cross section oriented as in Fig. 4. The axial scan was performed following the sequence from the positive to the negative surfaces to map a line from (x = 80 μm, z = 100 μm) to (x = 80 μm, z = 2500 μm) with a step of 3 μm (Dotted arrow 1 in Fig. 4). Note that the first z = 100 μm were not be mapped, to avoid the effect of the contamination of the surface by the resin used for embedding. Also, to avoid any instrumental laser-spectrometer drift over time the mapping was made in two steps, each with an experimental time of ~20 hours. The laser-spectrometer stability was checked at the beginning and the end of each mapping (after ~20 hours). The radial mapping was performed close to the centre of the pellet following the sequence from (x = 50 μm, z = 1300 μm) to (x = 1250 μm, z = 1300 μm), along the dotted arrow 2 of Fig. 4. Also in this case the first 50 μm from the edge were discarded because of contamination with the resin. All Raman spectra were normalised to the sum of their intensity 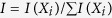. This permits to correct the Raman signal from instrumental effects like a change in the scattering volume, change in the z- focus, potential fluctuations of the laser power, etc.

The microstructure of the sintered samples was studied by optical and scanning electron microscopy (SEM, Philips XL 40) on fracture surfaces and polished and chemically etched surfaces. Etching was performed by a mixture of HNO_3_ and H_2_O in equal proportions at room temperature for 5 min. The grain size was measured by the line intercept method.

The temperature and current distributions within the pellet, as well as the Peltier heating and cooling at the graphite/UO_2_ interfaces, were evaluated by Finite Element Method (FEM) calculations using FlexPDE professional v6.37 [PDE Solutions Inc., Spokane Valley, WA, USA]. Since the thermal and electrical conductivity of uranium dioxide are largely dependent on the stoichiometry, a simplification was made and only the two extreme cases of UO_2.00_ and UO_2.16_ were considered. In addition, two different heights (3 mm and 1 mm) and three different temperatures (800 °C, 1000 °C and 1600 °C) were simulated. More details on the simulations are described in Appendix 1.

## Additional Information

**How to cite this article:** Tyrpekl, V. *et al*. On the Role of the Electrical Field in Spark Plasma Sintering of UO_2+x_. *Sci. Rep.* **7**, 46625; doi: 10.1038/srep46625 (2017).

**Publisher's note:** Springer Nature remains neutral with regard to jurisdictional claims in published maps and institutional affiliations.

## Supplementary Material

Supplementary Appendix

## Figures and Tables

**Table 1:** Uranium valences (%) and O/M ratio calculated from XANES spectra.

Sample	U(IV)	U(V)	U(VI)	O/M
1600 °C Conventional	100	0	0	2.00 (1)
1600 °C SPS	100	0	0	2.00 (1)
1000 °C SPS	76	24	0	2.12 (1)

**Table 2:** Stoichiometry of the surfaces of the UO_2_ pellets sintered in contact with the positive piston (top) and negative piston (bottom), calculated from XRD shifts.

Sample	O/M
800 °C SPS (positive surface)	2.14
800 °C SPS (negative surface)	2.03
1000 °C SPS (positive surface)	2.05
1000 °C SPS (negative surface)	2.00

The estimated error is ±0.01.
