# Supplementary material for: On the Role of the Electrical Field in Spark Plasma Sintering of UO2+x
Source: Sci Rep. 2017 Apr 19;7:46625. doi: 10.1038/srep46625 (PMC5395941; doi:10.1038/srep46625)
Supplement: Supplementary Appendix [file srep46625-s1.pdf]

Supplementary Information

On the Role of the Electrical Field in Spark Plasma Sintering of UO_{2+x}

*Vaclav Tyrpekl[a], Mohamed Naji[a], Michael Holzhäuser[a], Daniel Freis[a], Damien Prieur[a], Philippe Martin[b], Bert Cremer[a], Mairead Murray-Farthing[a], *Marco Cologna[a]*

a) European Commission, Joint Research Centre (JRC), Postfach 2340, 76125 Karlsruhe, Germany

b) CEA, DEN, DTEC, Centre d'études nucléaires de Marcoule, Bagnols-sur-Cèze F-30207, France

*Correspondence to marco.cologna@ec.europa.eu

Appendix 1

For the FE analysis, the geometry of the SPS setup was reproduced in a static two dimensional axisymmetric model (Fig. A1), considering the final sintered dimensions of the pellet. The UO_2 disks and pellets were simulated with both 1 mm and 3 mm thickness, respectively, and 6 mm diameter.

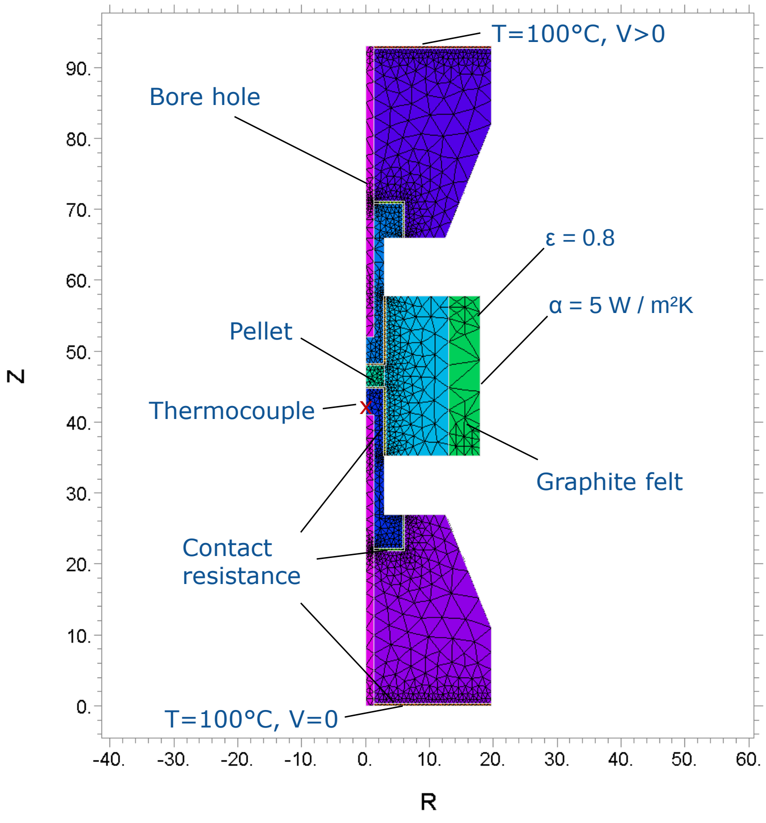

Fig. A1. Geometry considered for the Finite element model.

Only heat and current flows were simulated. No mechanical stresses or deformations were considered, and the simulations were performed for the final stage of sintering, not considering any extra deformation. Thermal-electrical coupling was simulated only via Joule heating. The governing

equations for electrical current flow, volumetric heating, solid phase heat transport and heat transfer to the surrounding furnace walls are summarised in Table A1.

Table A1. Governing equations of the FEM simulations.

THERMAL	ELECTRICAL
$\nabla \cdot (k\nabla T) + \dot{q}''' = 0$	$\nabla \cdot (g\nabla u) = 0$
k: Thermal conductivity $(W/mm\ K)$	g: Electrical conductivity (S/mm)
T: Temperature (K)	u: Voltage (V)
$\dot{q}'''$: Volumetric heat source (W/mm^3)	
	Coupling between electric and thermal equations
$\dot{q}'' = \sigma\varepsilon \cdot (T^4 - T_u^4) + \alpha \cdot (T - T_u)$	$\dot{q}''' = g(\nabla u)^2$
σ: Stefan – Boltzmann constant	
ε: Emmissivity (0.8)	
T_u: Environment temperature (30 °C)	
α: Heat transfer coefficient (5E-6 $W/mm^2\ K$)	

The heat transfer to the furnace walls and furnace atmosphere was simulated by thermal radiation (the emission coefficient of graphite was set to 0.8) and convection (heat transfer coefficient 5 10^{-6} W/mm² K). Electrical and thermal conductivity of the graphite were set to 160 S/mm and 0.15 W/mm K, respectively, according to the properties of SGL R7710 graphite at 1000°C. The furnace wall and atmosphere temperature was set to 30°C. The upper and lower graphite piston temperature was set to 100°C.

Power control was achieved by defining a global variable "voltage", which was controlled by a target temperature at the positon of the thermocouple, inside the bottom graphite piston 3 mm below the UO_2 pellet. The voltage at the bottom piston was set to 0, while the voltage at the upper piston was controlled by the global variable "voltage" Current was simulated as constant Direct Current (DC), and not as pulsed DC as in the real experiment.

Thermal conductivity (k) of UO_2 was simulated as function of temperature and stoichiometry. Simulations were performed on the two limiting cases of stoichiometric $UO_{2.00}$ and hyper-stoichiometric $UO_{2.16}$. The disk porosity was defined as 3% according to experimental results. The temperature dependent thermal conductivity of $UO_{2.00}$ and $UO_{2.16}$ were calculated according to Eq. A1 and A2, respectively[A1]:

$$k = \frac{1.158}{1000} \cdot \left(\frac{1-p}{1+2p}\right) \cdot \left(\frac{100}{7.548 + 17.692t + 3.614t^2} + \frac{6400}{t^{5/2}} \exp\left(\frac{-16.35}{t}\right)\right) \quad \text{(Eq. A1)}$$

$$k = \frac{1}{1000} \cdot \left(\frac{1-p}{1+2p}\right) \cdot \left(\frac{1}{0.0257 + 3.336x + (2.206 - 6.85x) \cdot \frac{t}{10}} + 1.158 \cdot \frac{6400}{t^{5/2}} \exp\left(\frac{-16.35}{t}\right)\right) \quad \text{(Eq. A2)}$$

where t is T/1000, with T temperature in K, p the porosity (0.03), and x the deviation from stoichiometry (0.016). The electrical conductivity (g) of $UO_{2.00}$ in S/mm was calculated as: [A2]

$$g = \frac{0.1}{47.5 \cdot T^{-1.4} \cdot \exp\left(\frac{1.06 \cdot 10^4}{T}\right)} \qquad \text{(Eq. A3)}$$

The electrical conductivity of hyperstoichiometric $UO_{2.16}$ was calculated by multiplying the conductivity of stoichiometric $UO_{2.00}$ in Eq. A3 by a factor of 50, according to data extrapolated from Ruello et al.[A3].

The model was calibrated by adjusting the effective thermal conductivity of the insulating felt (see Fig. A1) for power calibration, and by adjusting the contact resistance between the graphite electrodes and the graphite pistons as well as the contact resistance between the graphite electrodes and the disk for voltage and current calibration. By these adjustments the measured values during the SPS process were approximated in the simulation. Table A2 shows the measured and simulated values.

Table A1. Measured and simulated SPS data

	**SPS data**	**Simulation data**
**Temperature**	1000°C	1000°C
**Voltage**	5.44 V	3.70 V
**Current**	0.19 A	0.30 kA
**Power**	1.06 kW	1.10 kW

The Peltier power can be calculated by the material and temperature dependent Peltier coefficient and the current flowing through the disk.

$$\frac{Q}{t} = P_P = \pi I = \alpha T I \qquad \text{(Eq. A4)}$$

Where P_p is the Peltier power, π is the Peltier coefficient, α is the Seebeck coefficient and I the current going through the pellet derived from the FEM simulations. The results of the calculations are given in Table A2.

Table A2. Calculated Peltier power at the graphite/UO_2 interfaces

**Case**	**Peltier power (3 mm disk)**	**Peltier power (1 mm disk)**
**800°C, UO_2**	8 mW	13 mW
**800°C, $UO_{2.16}$**	23 mW	123 mW
**1000°C, UO_2**	6 mW	18 mW
**1000°C, $UO_{2.16}$**	262 mW	438 mW

Appendix 2

Relationship between Raman intensity and stoichiometry

A typical Raman spectrum of $UO_{2.00}$ acquired with the 647 nm laser in the 200 – 1300 cm^{-1} spectral range shows a peak at 446 ± 1 cm^{-1}, attributed to the stretching motion of the eight oxygen atoms around U atoms, and a strong band at 1150 cm^{-1}.[A4] The latter originates from the scattering of two longitudinal optical (LO) phonons having the same magnitude and opposite momentum ($\vec{Q_i}$) and is typically referred as the 2LO peak. It is observed in defect free $UO_{2.00}$ because it satisfies the Raman fundamental selection rule ($\sum_i Q_i = 0$). Another mode at 575 cm^{-1} is attributed to the LO(Γ) phonon and would be Raman forbidden in a perfect Fm-3m crystal. This mode is activated by defects via a multi-phonon process, as suggested in recent literature,[A4] and is observed in hyperstoichiometric UO_2 (Fig. A2). The ratio of intensity of these modes (at 575 and 1150 cm^{-1}) was shown to be very sensitive to electronic doping, in particular oxygen defects. Concretely, as the concentration of oxygen interstitials increases, the intensity of the LO mode increases, while the 2LO decreases up to certain oxygen concentration (2.25, U_4O_9) where it disappears completely [A5, A6]. The I(LO/2LO) can be used to probe the stoichiometry of UO_{2+x} samples.

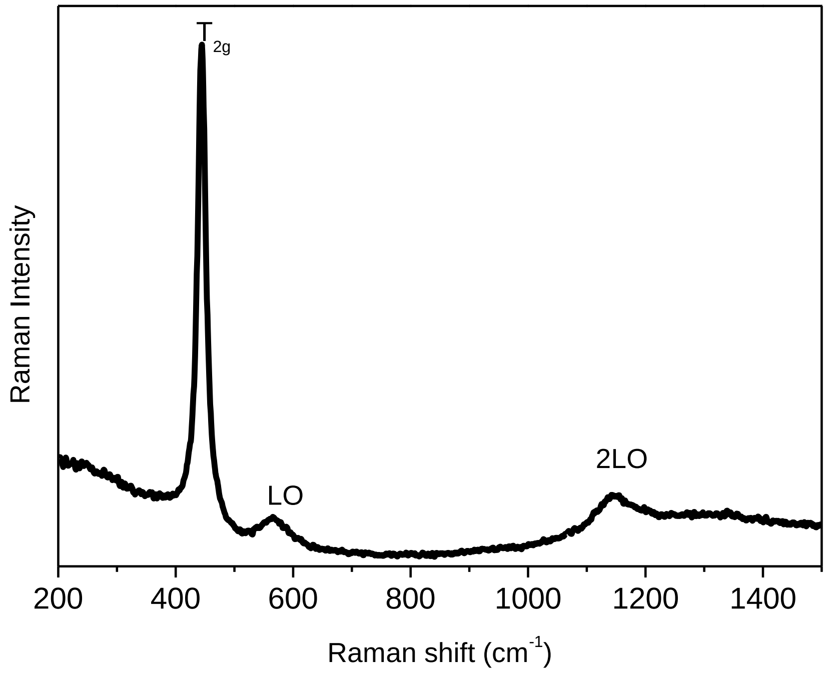

Fig. A2. A typical Raman spectrum of UO_{2+x} acquired with the 647 nm laser in the 200 – 1300 cm^{-1} spectral range for this work.

UO_2 is considered as Mott-Hubbard insulator where charges are completely localized and excited states are separated by the Coulomb energy U prohibiting double occupancy in the Hubbard model[A7-A9]. Electronic defects in uranium dioxide move through the crystal only by phonon assisted hopping (polaron formation) [A7-A9]. This mechanism enhances phonon assisted optical transitions in the range of energies below the band gap with a transition probability that is related to the distribution of the LO phonons. The increase of electronic defects, especially with an electron doping, results in a strengthening of the coupling force λ between LO phonons and the small-polarons which enhance the Raman intensity.

Component analysis

To overcome intensity effects of the Raman spectra, component analysis was performed on the vertical set of data. To reveal change in the LO and 2 LO modes, only the spectral region from 500 cm^{-1} to 1300 cm^{-1} was used. Raman maps were split in two matrices (z = 100 to 1300 and z= 1300 to 2500 µm) each consisting of 403 spectra, and component analysis was applied using discrete component least squares method (discrete component least squares fitting of the unknown data to a linear combination of specified component spectra) to decompose each spectrum into components from a set of reference spectra:

$$I(X_i, k_j) = \sum_{k=1}^{N} C_k(X_i) S_k(k_j) \qquad \text{(Eq. A5)}$$

where $S_k(k_j)$ are reference spectra and $C_k(X_i)$ their relative contribution. N is the number of reference spectra. Here, only two references spectra contribute to the original data (N=3). The first one, main spectrum, is issued from the scanned zones and can be considered as typical of averaged spectrum of the Raman data. The second one is representative of the change in the spectra form of the mode at 575 cm^{-1} together with that at 1150 cm^{-1}. The obtained decomposition of Raman spectra is drawn on Fig. A3 (a, b) and their corresponding scores in Fig A3 (c, d). Interestingly the contribution of the second component (PC2) to the Raman spectra follows almost the same trend observed in the change of α (Fig. A3 (c,d)). Thus, giving a strong support to the change of O/M ratio as revealed by the I(LO/2LO) .

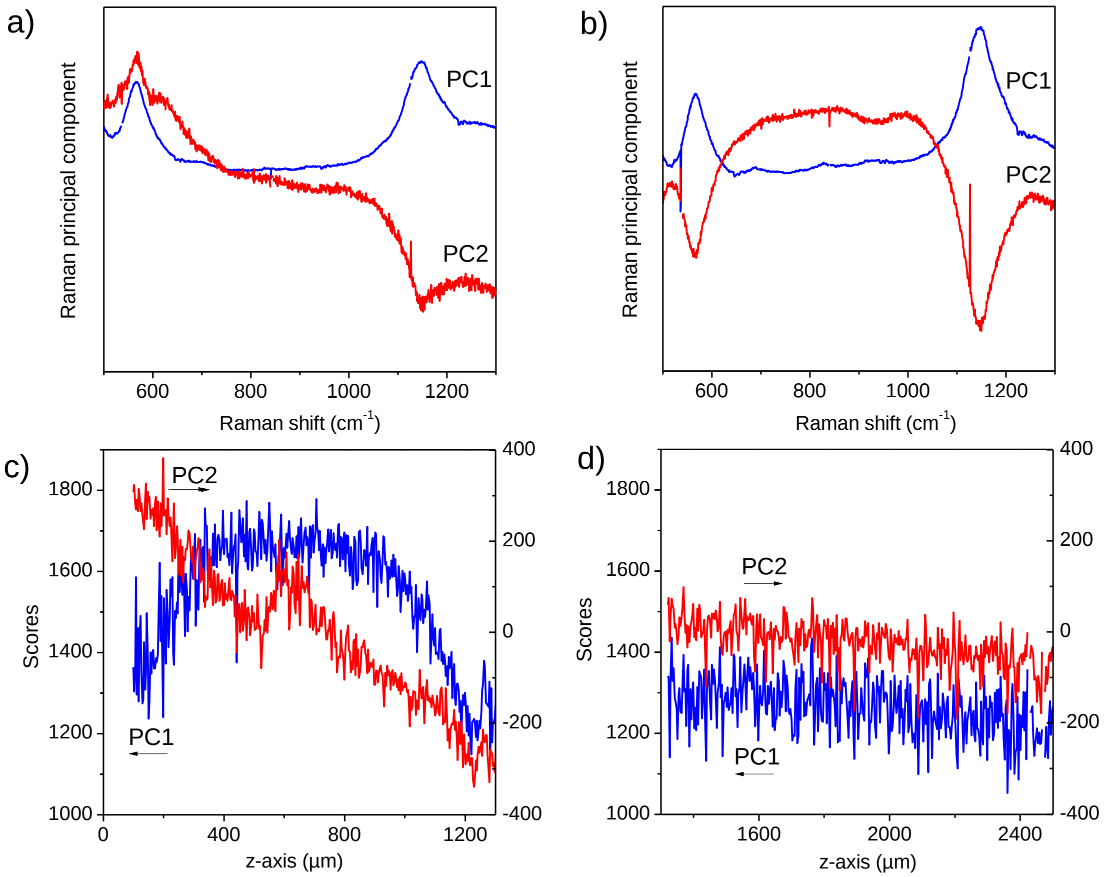

Figure A3. Component analysis performed in the Raman data acquired during the axial mapping. Principal components PC1 and PC2 (a) and (b), and their corresponding scores (c) and (d), along z = 100 -1300 µm (a and c) and along z=1300 - 2500 µm (b and d).

Supplementary References

A1. Carbajo, J. J., Yoder, G. L., Popov, S. G. & Ivanov, V. K. A review of the thermophysical properties of MOX and UO_2 fuels. J. Nucl. Mater. **299**, 181-198 (2001).

A2. Freund, D. & Schikarski, W. Der Direkt Elektrisch Geheizte UO-Brennstab. KFK-1031 (1970).

A3. Ruello, P., Petot-Ervas, G., Petot, C. & Desgranges, L. Electrical conductivity and thermoelectric power of uranium dioxide. J. Am. Ceram. Soc. **88**, 604-611 (2005).

A4. Livneh, T & Sterer, E. Effect of pressure on the resonant multiphonon Raman scattering in UO_2. Phys. Rev. B. **73** 085118 (2006).

A5. Manara, D. & Renker, B. Raman spectra of stoichiometric and hyperstoichiometric uranium dioxide. J. Nucl. Mater. **321**, 233-237 (2003).

A6. Naji. M. et al. Raman Scattering from Decoupled Phonon and Electron States in NpO_2. J. Phys. Chem. C. **120**, 4799- 4805 (2016).

A7. Conradson, S. D. et al. Possible Bose-condensate behavior in a quantum phase originating in a collective excitation in the chemically and optically doped Mott-Hubbard system UO_{2+x}. Phys. Rev. B. **88**, 115135 (2013).

A8. Conradson, S. D. et al. Possible Demonstration of a Polaronic Bose-Einstein(-Mott) Condensate in $UO_{2(+x)}$ by Ultrafast THz Spectroscopy and Microwave Dissipation. Sci. Rep. **5**, 15278 (2015).

A9. Yin, Q. & Savrasov, S. Y. Origin of low thermal conductivity in nuclear fuels. Phys. Rev. Lett. **100**, 225504 (2008).
